# Supplementary material for: Adipophilin expression is an independent marker for poor prognosis of patients with triple-negative breast cancer: An immunohistochemical study
Source: PLoS One. 2020 Nov 17;15(11):e0242563. doi: 10.1371/journal.pone.0242563 (PMC7671517; doi:10.1371/journal.pone.0242563)
Supplement: S1 Table — (DOCX) [file pone.0242563.s001.docx]

| **S1 Table. Clinical characteristics of patients with triple-negative breast cancer** | | | |
| --- | --- | --- | --- |
| Factors |  | n | % |
| Total |  | 61 |  |
| **Age (years)** | Median (range) | 58 (31–93) |  |
| **Menopausal status** | Premenopausal | 9 | 14.8 |
|  | Postmenopausal | 51 | 83.6 |
|  | Unknown | 1 | 1.6 |
| **Body mass index** | Median (range) | 23.3 (16.2–32.2) |  |
| **Tumor size (mm)** | Median (range) | 20 (2–55) |  |
| **Pathological stage** | I | 25 | 41.0 |
|  | IIA | 22 | 37.7 |
|  | IIB | 6 | 8.2 |
|  | IIIA | 4 | 6.6 |
|  | IIIB | 3 | 4.9 |
|  | IIIC | 1 | 1.6 |
| **Lymph node status** | Positive | 14 | 23.0 |
|  | Negative | 33 | 54.0 |
|  | Not tested | 14 | 23.0 |
| **Lymphatic invasion** | Positive | 53 | 86.9 |
|  | Negative | 8 | 13.1 |
| **Venous invasion** | Positive | 37 | 60.7 |
|  | Negative | 24 | 39.3 |
| **Nottingham histological grade** | 1 | 2 | 3.3 |
|  | 2 | 27 | 44.3 |
|  | 3 | 32 | 52.4 |
| **Ki-67 labeling index** | High | 26 | 42.6 |
|  | Low | 33 | 54.1 |
|  | Not evaluated | 2 | 3.3 |
| **Adjuvant chemotherapy** | Administered | 35 | 57.4 |
|  | Not administered | 23 | 37.7 |
|  | Undetermined | 3 | 4.9 |
