# Supplementary figures and images for: Adipophilin expression is an independent marker for poor prognosis of patients with triple-negative breast cancer: An immunohistochemical study
Source: PLoS One. 2020 Nov 17;15(11):e0242563. doi: 10.1371/journal.pone.0242563 (PMC7671517; doi:10.1371/journal.pone.0242563)

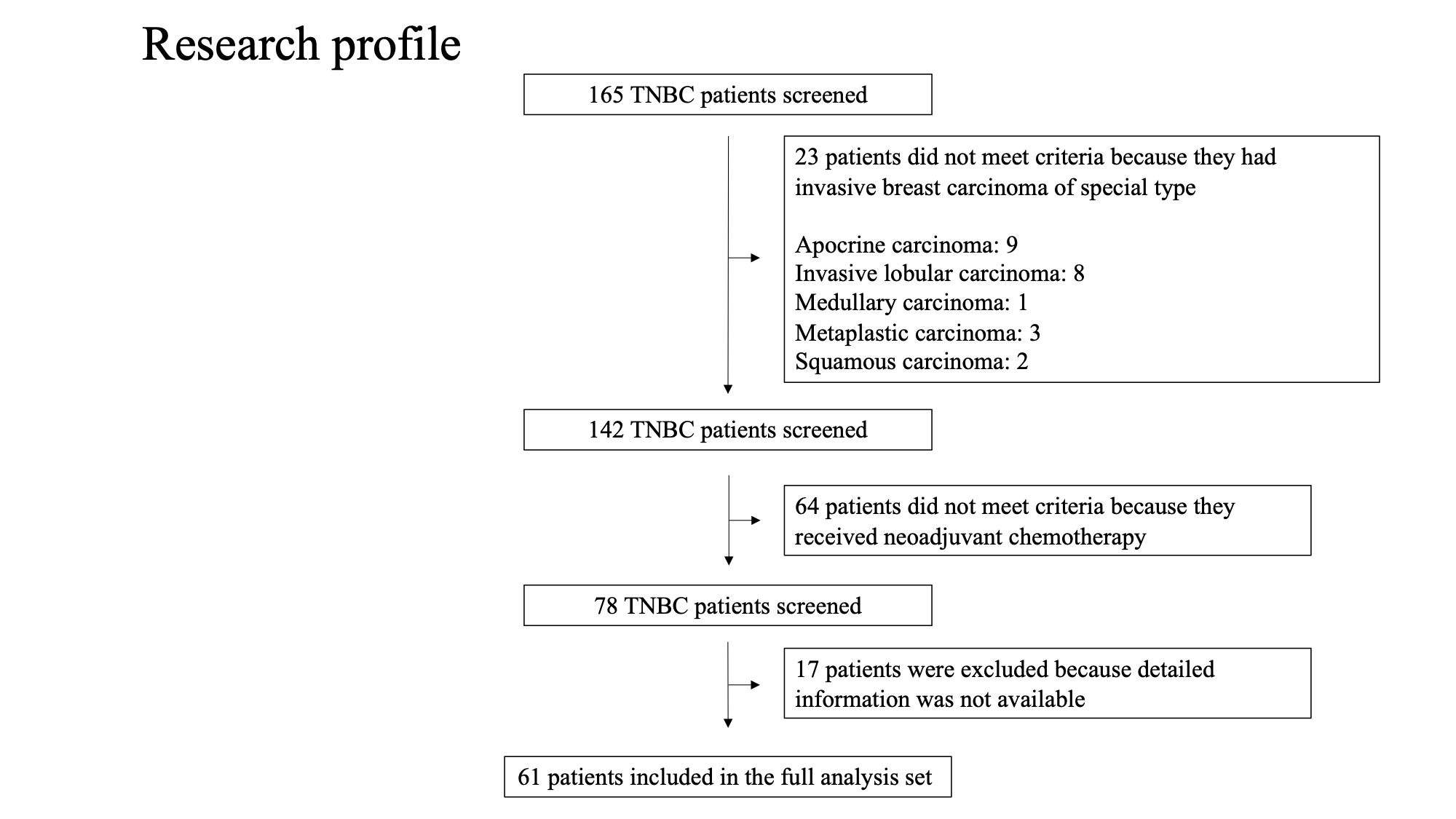

Supplement: S1 Fig — (TIFF) [file pone.0242563.s002.tiff]
