# Supplementary material for: Adipophilin expression is an independent marker for poor prognosis of patients with triple-negative breast cancer: An immunohistochemical study
Source: PLoS One. 2020 Nov 17;15(11):e0242563. doi: 10.1371/journal.pone.0242563 (PMC7671517; doi:10.1371/journal.pone.0242563)
Supplement: S1 Data — (PDF) [file pone.0242563.s003.pdf]

第 1 版

2020 年 1 月 15 日

乳癌摘出術を受けられた患者さんへ（臨床研究に関する情報）

当院では、以下の臨床研究を実施しております。この研究は、摘出標本と診療情報等を使って行います。このような研究は、文部科学省・厚生労働省の「人を対象とした医学系研究に関する倫理指針」の規定により、研究内容の情報を公開することが必要とされております。なお、この臨床研究は関西医科大学附属病院研究倫理審査委員会の審査を受け、研究方法の科学性、倫理性や患者さんの人権が守られていることが確認され、病院長の許可を受けています。この研究に関するお問い合わせなどがありましたら、以下の「問い合わせ先」へご照会ください。

《研究課題名》乳癌における adipophilin 発現の臨床病理学的意義の解明

《研究機関名・研究責任者》　関西医科大学附属病院・病理診断科　講師　石田光明

《研究の目的》乳癌において脂肪関連蛋白であるアディポフィリン（adipophilin）の発現が予後因子となるか、さらにアディポフィリンの発現と糖代謝やグルタミン代謝酵素の発現との相関を解明する

《研究期間》研究許可日～2023 年 12 月 31 日

《研究の方法》

●対象となる患者さん

乳腺腫瘍の患者さんで、2006 年 1 月 7 日から 2018 年 12 月 31 日の間に乳癌摘出術を受けた方

●研究に用いる試料・情報の種類

情報：診断名、年齢、性別、身体所見、検査結果（画像検査）等

試料：手術で摘出した組織のパラフィンブロック

《この研究に関する情報の提供について》

この研究に関して、研究計画書や研究に関する資料をお知りになりたい場合は、他の患者さんの個人情報や研究全体に支障となる事項以外はお知らせすることができます。

《この研究での検体・診療情報等の取扱い》

お預かりした検体や診療情報等には匿名化処理を行い、患者さんの氏名や住所などが特定できないように安全管理措置を講じたうえで取扱っています。

《本研究の資金源・利益相反について》

この研究は外部の企業等からの資金の提供は受けておらず、研究者が企業等から独立して計画して実施しているものです。したがって、研究結果および解析等に影響を及ぼすことは無く、患者さんの不利益につながることはありません。また、この研究の研究責任者および研究者は「関西医科大学利益相反マネジメントに関する規程」に従って、利益相反マネジメント委員会に必要事項を申請し、その審査と承認を得ています。

＊上記の研究に利用することをご了解いただけない場合は以下にご連絡ください。

《問い合わせ先》

関西医科大学附属病院　病理診断科　　担当医師　講師　石田光明

大阪府枚方市新町2丁目3-1

電話　072-804-2794　　FAX　072-804-2960
